# Force-induced melting of DNA—evidence for peeling and internal melting from force spectra on short synthetic duplex sequences

**DOI:** 10.1093/nar/gku441

**Published:** 2014-05-16

**Authors:** Niklas Bosaeus, Afaf H. El-Sagheer, Tom Brown, Björn Åkerman, Bengt Nordén

**Affiliations:** 1 Department of Chemical and Biological Engineering, Chalmers University of Technology, Gothenburg S41296, Sweden; 2 School of Chemistry, University of Southampton, Southampton, SO17 1BJ, UK

## Abstract

Overstretching of DNA occurs at about 60–70 pN when a torsionally unconstrained double-stranded DNA molecule is stretched by its ends. During the transition, the contour length increases by up to 70% without complete strand dissociation. Three mechanisms are thought to be involved: force-induced melting into single-stranded DNA where either one or both strands carry the tension, or a B-to-S transition into a longer, still base-paired conformation. We stretch sequence-designed oligonucleotides in an effort to isolate the three processes, focusing on force-induced melting. By introducing site-specific inter-strand cross-links in one or both ends of a 64 bp AT-rich duplex we could repeatedly follow the two melting processes at 5 mM and 1 M monovalent salt. We find that when one end is sealed the AT-rich sequence undergoes peeling exhibiting hysteresis at low and high salt. When both ends are sealed the AT sequence instead undergoes internal melting. Thirdly, the peeling melting is studied in a composite oligonucleotide where the same AT-rich sequence is concatenated to a GC-rich sequence known to undergo a B-to-S transition rather than melting. The construct then first melts in the AT-rich part followed at higher forces by a B-to-S transition in the GC-part, indicating that DNA overstretching modes are additive.

## INTRODUCTION

**Figure 1.**
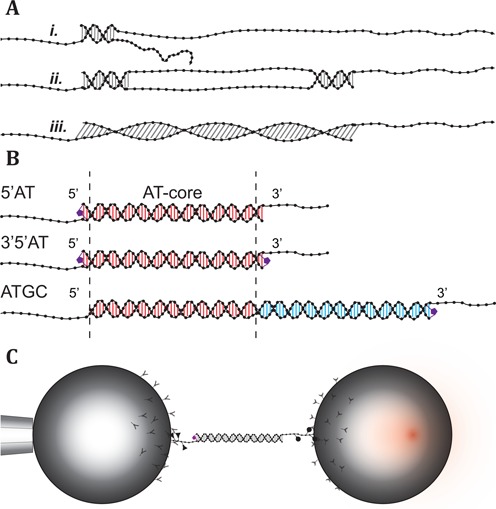
Overstretching mechanisms, DNA constructs and experimental setup. (**A**) The three proposed mechanisms for overstretching. (*i.*) Peeling, where the DNA progressively melts from free ends. (*ii.*) Internal melting, where the melting is initiated in regions of low stability forming ‘bubbles’. (*iii.*) B-to-S transition, where the base-pairing remains intact as the duplex is extended into a longer form. (**B**) Schematic description of the DNA constructs and the covalent inter-strand linkage. 5′AT: 64 bp with a terminal inter-strand cross-link. The inter-strand linker is formed by joining alkyne and azide modified bases on opposite strands together using the Cu^+^-catalyzed click reaction. 3′5′AT: 64 bp with one inter-strand linker at each end of the duplex region. ATGC: 122 bp duplex region consisting of the AT-core sequence shared with 5′AT and 3′5′AT, and a previously studied single-clicked GC-rich sequence (23). For bead attachment, each construct is extended in the 3′-ends with single-stranded DNA containing digoxigenin or biotin modified bases. (**C**) Experimental setup. The DNA constructs are tethered to the streptavidin and anti-digoxigenin coated beads by biotin and digoxigenin modified bases incorporated in the single stranded handles, respectively. One of the beads (left) is immobilized by suction onto a pipette, while the other (right) is manipulated by the optical trap.

Mechanical deformation of DNA is involved in many biological processes. It occurs, for example, during repair, compaction and regulation of the activity of the genome (1–3). Detailed knowledge of the mechanical properties of DNA is, therefore, necessary for fully understanding these fundamental processes. The deformation may not only be a structural or topological hurdle that the system has to overcome in order to reach the next step of the biological process, it may itself constitute a regulatory or discriminatory function. For example, in its complex with recombinase protein RecA, the DNA strands involved in genetic recombination are stretched in a way that has been proposed to play a role in the testing and execution of rehybridization (1). How DNA responds to longitudinal stress has been studied on a single-molecule level for over two decades (4). The perhaps most striking feature in the dynamics of double-stranded (ds) DNA under tension is the overstretching transition. During this transition, the DNA undergoes a conformational change where its extension increases up to 70% without complete strand dissociation. Since the discovery of the overstretching transition (5,6) much effort has been put into understanding the structural changes that occur during the transition (7–25). The current consensus regarding the fate of the dsDNA during overstretching suggests that probably three different structural changes may account for the increase in contour length (Figure 1A). Two of the models involve partial or complete strand separation (melting) induced by the applied force where the base-pairing between the two strands in the duplex is broken. During a peeling transition (*i.*), the base-pairing is progressively broken starting from a free end of the duplex. This process leaves one strand carrying the full tension while the complementary strand is relaxed. In the second melting model (*ii.*), the strand separation is initiated locally inside the dsDNA and results in molten regions where base-pairing is lost separated by domains where the base-pairing is still intact. These internally melted regions have been referred to as ‘melting bubbles’ due to their similarity with thermally denatured DNA (7,14). In the third model (*iii.*), the base-pairing remains intact and the dsDNA unwinds to accommodate the overstretching (26–29). The detailed structure of this novel stretched state of dsDNA called S-form is however still unresolved. Conditions such as salt, pH, temperature and the DNA sequence determine to which extent these processes may be combined in response to an increase in the applied force. This static balance together with local dynamic fluctuations and transitions between the different states set the stage for a complex behavior. Most studies on the overstretching transition have been performed on DNA several thousands of base pairs (bp) long where combinations of these processes are likely to occur simultaneously and, therefore, may escape detection.

Here we use optical tweezers to study designed dsDNA oligonucleotides, 64–122 bp long with systematically varied sequences, in an effort to identify and isolate different mechanistic behaviors, focusing primarily on force-induced melting phenomena and their dependence on ionic strength. The potential occurrence of strand separation is detected by exposing the DNA to glyoxal during stretching. If base pairs are broken and become exposed to the solvent, the glyoxal forms a covalent adduct with guanine bases (9,22,23). The glyoxal adduct prevents base pairs from reforming with the complementary cytosine bases and thus inhibits rehybridization (30,31). Using click-chemistry we can also introduce inter-strand linkers designed to covalently seal one or both ends of the DNA duplex to selectively study both types of force-induced melting in the same sequence (23,32,33). By covalently cross-linking one end of a 64 bp long AT-rich duplex we can repetitively melt and rehybridize the sequence, switching between native B-form and a peeled state. With the addition of a second click-linker at the other end of the duplex the construct is instead reversibly transitioned into an internally melted state. Finally we study the peeling transition in a 122 bp long sequence containing the same AT-rich sequence concatenated to a GC-rich sequence that is known to overstretch into the S-form when studied on its own (23). The short and well-defined oligonucleotide duplex sequences allow the fine structure of the force spectrum to be observed, revealing features characteristic of different modes of melting and conformational states of extended DNA double helices. Our results confirm the presence of both peeling and melting bubble formation and also show that the two force-induced melting processes exhibit distinguishable thermodynamic and kinetic differences, behaviors that depend on salt concentration and on local sequence environment. The results also show that these melting processes may occur without detectable hysteresis and that the S-form state may not be accessible for all base compositions at room temperature.

## MATERIALS AND METHODS

### Design of DNA constructs

The three DNA constructs, schematically depicted in Figure 1B, each consist of a double-stranded region and single-stranded handles for bead attachment. Two of the constructs, 5′AT and 3′5′AT, have a double-stranded region of 64 bp while in the third construct, ATGC, the double-stranded region is 122 bp long (see Supplementary Figure S1 for details). The three DNA constructs all share a common 60 bp AT-rich (30% GC-content) core sequence. The constructs were designed (oligonucleotides from ATDBio, UK) with alkyne and azide modified bases at either or both ends of the duplex region so that that the strands could be covalently cross-linked using Cu^+^-catalyzed click-chemistry (0.01eq oligonucleotide, 1eq CuSO_4_ • 5H_2_O, 7eq tris-hydroxypropyltriazole, 10eq Na ascorbate, overnight at 22°C) (32,34,35). The formed inter-strand links connect two C5-modified pyrimidine bases on opposite strands diagonally from one modified base to the 5′-neighboring base on the complementary strand. The 5′AT construct has a linker in one end while the 3′5′AT has both ends of the double-stranded region sealed by inter-strand links. In the ATGC construct, the AT-rich core is positioned next to a GC-rich sequence previously studied by us (23) that has a GC-content of 60%. In the GC-rich end of the construct, an inter-strand linker site was also added that adds an additional two base pairs to the duplex sequence.

The handles were formed by extension of the 3′-ends using terminal transferase (New England Biolabs), prior to hybridization of the duplex, with a dUTP-biotin or dUTP-digoxigenin to dATP ratio of 1:10 (Digoxigenin-11-dUTP, Biotin-16-dUTP, Roche; see Supplementary Figure S2). The incorporated digoxigenin- or biotin-modified nucleotides allow attachment to polystyrene beads coated with digoxigenin antibodies or streptavidin, respectively. Streptavidin beads with a nominal diameter of 2.1 μm were purchased from Spherotech. Anti-digoxigenin beads of the same size were prepared in-house by cross-linking of anti-digoxigenin polyclonal antibodies (Roche) using dimethyl pimelimidate (Sigma) onto proteinG modified polystyrene beads from Spherotech.

### Instrument setup and measurements

In the experimental setup, two counter-propagating 150 mW, 845 nm diode lasers were used to form a single adjustable optical trap in order to capture one of the coated polystyrene beads. The other bead was immobilized by suction at the orifice of a pipette embedded in the fluidics chamber (see Figure 1C). The optical trap is steered in the plane perpendicular to the light axis using piezoelectric crystals by bending of the optical fibers guiding the laser beams. The position of the trap is measured by redirecting about 5% of the light intensity using a pellicle beam-splitter onto position-sensitive detectors (PSD), before the light is focused through a water-immersion objective lens (Olympus, UPLSAPO 60XW, NA 1.20) (36). The exiting light is collected by an identical objective lens that redirects it to PSDs where the force components in all three dimensions acting on the trapped bead are measured (37). The two laser beams have orthogonal polarizations that allow them to be separated using polarization selective beam-splitters and monitored individually (see Supplementary information for further information).

Measurements were performed in a buffered high (1 M NaCl) or low (5 mM NaCl) salt environment (10 mM Tris pH 7.4, 1 mM EDTA). In the glyoxal exposure experiments, 0.5 M glyoxal (Sigma-Aldrich) was included in the buffer, and the pH was adjusted to 7.9. Once a tether containing a single DNA construct was established between the two beads, experiments were performed by moving the trap at a constant velocity (50 nm/s) to increase or reduce the force applied to the 3′-ends of the construct. During a pull and relax cycle, the force was increased from a lower force limit to an upper force limit and then back down again at the same rate. The force limits were set to ensure a hybridized construct at the lower force limit and complete development of the studied transitions at the upper limit. Within the force range the effective stiffness of the construct and the optical trap is largely dominated by the trap, and thus the force is varied with an approximately constant loading rate. The mean loading rate for all experiments was 7.8 ± 0.57 pN/s (± 1 s.d.). Data were recorded at a frequency of 1 kHz, and are presented here as force versus trap position trajectories, which show the force acting on the captured construct as a function of the relative position of the optical trap. A consequence of this approach, where only the position of the optical trap is controlled combined with a relatively slow trap velocity, is that a sudden elongation of the construct is accompanied by a drop in the force as the bead relaxes toward the center of the trap. Reversal of the transition increases the force as the contracting molecule pulls the bead away from the trap center. Relative extensions were measured as the difference in trap position at a specific force. The temperature was measured inside the instrument close to the fluidics chamber. The mean temperature for all measurements was 22.8 ± 0.6°C.

## RESULTS

Mechanical stretching experiments were performed on a 60 bp AT-rich oligonucleotide in three synthetic DNA constructs (Figure 1B). The construct 5′AT where the AT-rich sequence is covalently sealed at one end by a click-linker was used to perform repeated melting experiments on one and the same molecule, the purpose of the linker being to prevent the melted construct from irreversibly falling apart. The construct covalently sealed at both ends (3′5′AT) was used to investigate if the same AT sequence can melt internally, rather than by peeling of the strands from the ends of the duplex. Thirdly, the same AT sequence was combined with a previously studied GC-rich sequence into a single synthetic construct (ATGC), with the aim to investigate the melting behavior of the AT sequence when integrated into a longer native DNA molecule.

### Inter-strand linkers allow repetitive melting experiments on the same DNA molecule

**Figure 2.**
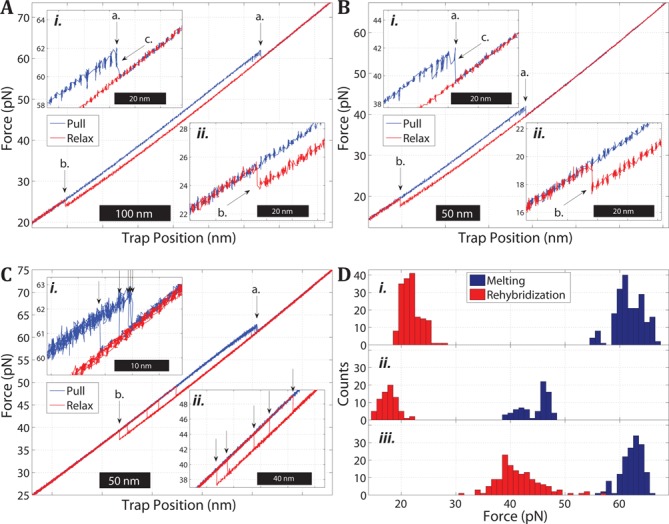
Melting and rehybridization of the AT-rich oligonucleotide in the single (5′AT) or double-clicked form (3′5′AT). (**A**) Pull and relax cycle of a 5′AT molecule in 1 M NaCl showing a melting transition at about 62 pN (a. and inset *i.*) during pulling (force is increased) and rehybridization (b. and inset *ii.*) at 24 pN during relaxation (force is decreased). Prior to the melting transition the molecule displays a region of reversible extension and contraction (c. in inset *i.*) likely due to partial melting of the double-stranded region. This intermediate state is observed for many but not all transitions and always coexists with the B-form state at any given trap position. (**B**) Pull and relax cycle of a 5′AT molecule in 5 mM NaCl. The melting force is reduced to 42 pN (a. and inset *i*) and the rehybridization force (b. and inset *ii.*) is also reduced by the decreased salt concentration but to lesser extent. The same type of bistability as in high salt is observed prior to complete melting of the duplex (c.). (**C**) Multiple pull (blue) and relax (red) cycles of a 3′5′AT molecule in 5 mM NaCl showing the melting (a. and inset *i*) and rehybridization (b. and inset *ii.*) transitions. The arrows in the insets mark the variation in melting and rehybridization forces within the same molecule during repeated cycles. (**D**) Histograms of melting (blue) and rehybridization (red) forces for (*i.*). Single-clicked 5′AT in 1 M NaCl, (*ii.*) 5′AT in 5 mM NaCl and (*iii.*) double-clicked 3′5′AT in 5 mM NaCl.

Figure 2A shows a typical pull and relax cycle of a single-clicked 5′AT molecule in 1 M NaCl. The applied force is plotted as a function of the trap position during the extension (blue) and relaxation (red) of the DNA construct. During pulling, the molecule extends abruptly at about 62 pN (a.), observed as a distinct drop in force as the bead moves toward the center of the optical trap (inset *i.*). During the relaxation part of the cycle, the molecule abruptly becomes shorter (increase in force) at about 24 pN (b.). The hysteresis observed during the relaxation indicates that the 5′AT construct is melted at high force as supported by glyoxal experiments (23), most likely by a force-induced peeling transition where all base pairs in the initial B-form state are disrupted. Inset *ii.* shows that during the relaxation, the measured force returns to the previous pulling curve, strongly indicating that the 5′AT construct has hybridized to reform the B-form duplex. Notably, non-clicked AT molecules were observed to fall apart irreversibly, so the click-linker in 5′AT was essential for keeping the construct together when all base-paring is lost during the melting. The possibility to repeat pull/relax cycles on the same molecule, despite melting, was used to collect data on the melting force *F_m_*, and corresponding extension calculated as the distance between the B-form and the melted state at *F_m_*. The mean value (*n* = 16 molecules) for *F_m_* was 61.5 ± 2.62 pN (± 1 s.d.) and the mean extension was 14.7 ± 0.99 nm (0.230 ± 0.015 nm/bp). The average rehybridization force *F_r_* during relaxation was 21.7 ± 1.27 pN and the corresponding contraction was 13.4 ± 0.59 nm (0.209 ± 0.009 nm/bp).

Close inspection of inset *i.* in Figure 2A shows that the irreversible melting (a.) is preceded by a transition at a lower force at which the construct jumps reversibly back and forth between the initial B-form and a longer state (c.). This intermediate always appeared in conjunction with the B-form and was never observed as an isolated state, and thus likely corresponds to a partial melting of the helix induced by the pulling, before the construct fully melts.

### Lower ionic strength decreases the melting force

Figure 2B shows a pull–relax cycle of 5′AT in 5 mM NaCl. The low salt behavior is similar in nature to the behavior at 1 M NaCl (Figure 2A), including the partially melted intermediate, but the values of the transition forces are overall lower. The average force required to melt the construct decreases by approximately 18 pN to 43.6 ± 2.79 pN (*n* = 12) while the average rehybridization force decreases by about 4.4 pN to 17.3 ± 0.98 pN. Figure 2D shows histograms of the measured melting and rehybridization forces for 5′AT at high (*i.*) and low salt (*ii.*). The ionic strength is seen to have a significant impact on the force-induced melting of short oligonucleotides, as has been observed previously for long DNA (10,18), but the force required for melting is reduced more strongly than for the rehybridization.

### The AT-rich sequence melts internally when sealed at both ends

**Figure 3.**
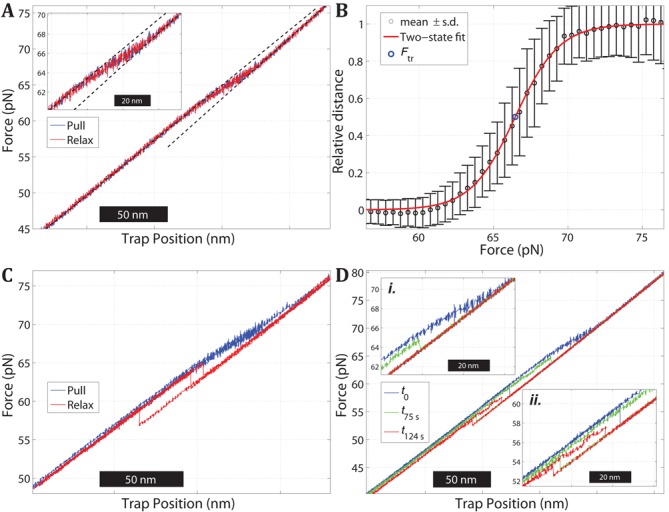
Stretching of a double-clicked 3′5′AT construct in 1 M NaCl. (**A**) Plot of force versus trap position during one cycle of pulling (blue) and relaxation (red). The DNA duplex exhibits a continuous transition over a broad force interval (60–70 pN) with no detectable hysteresis between the pulling and relaxation parts of the cycle. Dashed lines show least-mean-square linear fits to the force versus distance data below and above the transition region. (**B**) Pooled data from nine pull and relax cycles of the molecule in panel (A) showing the relative extension of the duplex versus the applied force. Experimental data (circles with error bars ± 1 s.d.) and fit to a two-state model (red curve) to estimate the force *F*_tr_ at the midpoint of the transition (see Supplementary information). (**C**) Multiple pull and relax trajectories of a 3′5′AT molecule exhibiting hysteresis during the relaxation. Fifteen out of 31 studied 3′5′AT molecules had one or several relaxation curves of this hysteretic type. (**D**) Pull experiments on a 3′5′AT molecule in the presence of 0.5 M glyoxal (1 M NaCl, pH 7.9) before and after reaction. The blue curve is the pull trace before the DNA was exposed to glyoxal, and shows how the molecule is gradually extended as in panel (A). The molecule was then kept at 74 pN in order to expose the construct to the glyoxal for certain period of time, after which the force was reduced to 20 pN (relaxation curve not shown) and a new pulling experiment was performed to evaluate the effect of the glyoxal. The green and red curves show the pulling of the same molecule after an exposure time of 75 and 124 s, respectively. The transition force is reduced to about 65 pN (green curve in inset *i*.) and 57 pN (red curve in inset *ii.*), respectively, indicating the formation of glyoxal adducts.

Figure 3A shows a pull and relax cycle of a double-clicked 3′5′AT molecule in 1 M NaCl. During pulling (blue), the molecule gradually extends over a force interval between 60 pN and 70 pN with no discernible intermediates, and during the relax part of the cycle (red) it does not show the hysteresis that is seen with the single-clicked 5′AT (Figure 2A). However, about half of the studied 3′5′AT molecules (15 out of 31) exhibited the alternative rehybridization behavior shown in Figure 3C. During pulling (blue), the molecule extends in the same gradual manner as in Figure 3A, but during relaxation (red) it displays a clear hysteresis and returns to the B-form in one abrupt step rather than showing the reversible relaxation over an extended force range as was seen in Figure 3A. Importantly, both relaxation pathways appear available to a given 3′5′AT molecule as they could be observed in one and the same molecule when it was monitored over several pull–relax cycles; the hysteretic case was observed in 34 out of 155 relax curves.

The hysteresis in Figure 3C shows that the 3′5′AT construct melts by force. Whether the non-hysteretic type of transition in Figure 3A also involves broken base pairs was further investigated by probing the sensitivity to glyoxal, previously used to confirm that the 5′AT oligonucleotide in Figure 2 melts at high force while a GC-rich oligonucleotide of the same length remains base paired (23). Figure 3D shows the results when a 3′5′AT molecule was subjected to repetitive pulling experiments in the presence of 0.5 M glyoxal (added to the 1M NaCl buffer) in order to monitor how the molecule is affected by glyoxal in the extended state. The blue curve shows that during the first cycle the molecule exhibits the same pulling behavior as in the absence of glyoxal (Figure 3A), as is expected since the extended state of the molecule has then not yet been exposed to glyoxal. The green curve shows the stretching behavior of the same molecule after it had been exposed to glyoxal for 75 s in the fully extended state. The reduced transition force (inset *i.*) indicates that glyoxal has formed covalent adducts, meaning that base pairs are disrupted in the extended state because the glyoxal adducts prevent rehybridization of the modified bases and thus lowers the melting force. A melting behavior is supported by the observation that the effect of glyoxal is accentuated if the extended state is exposed for a longer time (124 s in red curve; inset *ii.*).

The glyoxal experiments show that the double-clicked 3′5′AT melts under tension, and that the melting must occur internally in the duplex (Figure 1C) because the peeling mechanism is prevented by the covalent seals in both ends of the construct (Figure 1A). The gradual nature of the transition (evidenced from Figure 3A and C) suggests a low degree of cooperativity compared to the abrupt melting of 5′AT construct observed in Figure 2A. The midpoint force (*F*_tr_) and degree of cooperativity in the 3′5′AT overstretching process was estimated by using the data in Figure 3A to fit a two-state model intended to describe the transition from an initial B-form state to the fully stretched final state. The extent of the reaction was evaluated by calculating the relative distance of each data point to the linear force versus position behaviors below and above the transition region (dashed lines in Figure 3A). The resulting fit to the two-state model (Figure 3B) gives a mean value for *F*_tr_ of 67.8 ± 1.01 pN (*n* = 31), and a cooperative length (see Supplementary information) of 2.6 ± 0.4 nm (16 bp) that is short compared to the length of the 60 bp AT sequence (20.4 nm in the B-form). The calculated extension of the 3′5′AT molecule at the midpoint *F*_tr_ of the transition is 10.6 ± 1.26 nm (0.166 ± 0.020 nm/bp).

### Internally melted DNA rehybridizes via two pathways

Figure 2C shows the stretching behavior of a 3′5′AT molecule at 5 mM NaCl. The 3′5′AT duplex melts in a single step at low salt, in contrast to the gradual melting observed at high salt (Figure 3A and 3C), in a manner more similar to the melting behavior of the single-clicked construct 5′AT at both high and low ionic strength (a. in Figure 2A and B). During relaxation, the 3′5′AT molecules exhibit hysteresis and rehybridize in a single step at low force. The arrows during melting (inset *i.*) and rehybridization (inset *ii.*) show that both transitions occur in a relatively wide force range, and the distributions for 14 molecules are shown in Figure 2D (*iii.*). The average force (*n* = 14) was 61.0 ± 2.27 pN for melting and 41.1 ± 4.88 pN for rehybridization. Both values are considerably higher than for 5′AT at 5 mM NaCl (Figure 2D *ii.*) as is expected from the enhanced duplex stability toward melting when both ends are sealed (23). The average extension during melting was 9.30 ± 0.85 nm (0.145 ± 0.013 nm/bp) and the contraction during rehybridization was 11.7 ± 0.47 nm (0.183 ± 0.007 nm/bp).

Notably, the double-clicked 3′5′AT construct rehybridizes in two manners. At low salt (Figure 2C), all studied molecules (*n* = 15) exhibit hysteresis during the relaxation whereas at high salt only about half of the molecules do (15 of 31; Figure 3C) while the rest instead rehybridize reversibly in all trajectories (Figure 3A). The different rehybridization modes of 3′5′AT could possibly be due to the construct attaining different molten states that relax differently. However, two observations indicate that the extended state at high force is one and the same, most likely a fully molten duplex. The length of the melted 3′5′AT construct is the same in the presence and absence of hysteresis because the measured extension during the transition from the B-form is the same (the difference being 0.1 ± 1.1 nm). Secondly, we observe no additional transition during the pulling phases that precede the hysteretic cases which otherwise could indicate a different type of melted state. In conclusion, the gradual melting in Figure 3A and C results in a 3′5′AT melted state that can rehybridize via different pathways, one of which with high enough activation energy to cause a measurably slow restoring of the base-pairing that is observed as a hysteresis. One clue to the difference in the mechanisms is that the barrier is enhanced at low ionic strength because the reversible type of rehybridization is only observed at high salt (Figure 3A). This suggests that the barrier involves the electrostatic repulsion between the strands. An alternative explanation, that some force-induced change in the linker conformation could be the cause of the delayed rehybridization, seems unlikely since the click-links are uncharged and should not be sensitive to salt change. In the more probable scenario, the barrier involves the DNA itself, electrostatic repulsion between phosphates on the same and/or opposite strands preventing the strands at high force to adopt the helical twist of the B-form.

### ATGC extends in two steps under tension

The melting behavior of the native AT-rich sequence (without the covalent linkers used in 5′AT and 3′5′AT) was studied in the ATGC construct, where the AT-part is connected to a 62 bp GC-rich sequence in a single synthetic 122 bp DNA molecule. The GC-part by itself does not melt under force in high salt (23), but in case the ATGC combination still undergoes force-induced melting the construct was designed with a click-linker in the GC-part (Figure 1B) to prevent it from falling apart.

**Figure 4.**
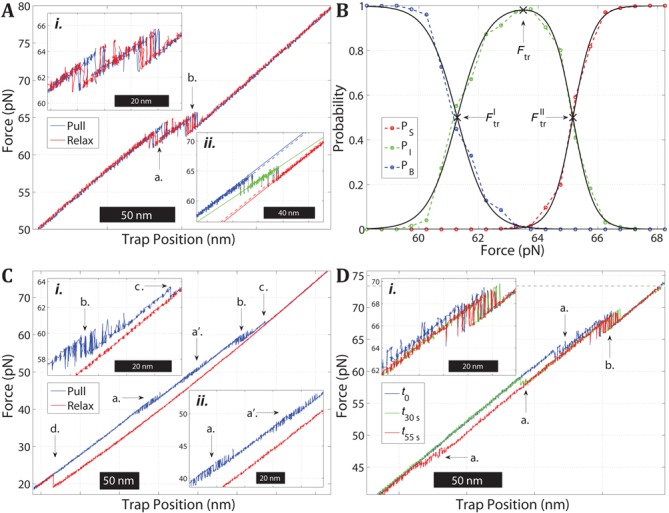
Force-induced melting and rehybridization of the ATGC construct. (**A**) Force versus trap position trajectories during multiple pull–relax cycles on an ATGC molecule in 1 M NaCl. The two distinct transitions (a. and b.) are reversible. Inset *i.* shows that the two transitions are well separated in force and that both exhibit bistability. Inset *ii.* shows a single pull trajectory for the same molecule and the linear fits (dashed) used to assign the data points to the three states at low (blue), intermediate (green) and high force (red). (**B**) Population probabilities of the three states as a function of force for the molecule in (A), based on pooled data from the pull and relax trajectories. The fitted curves (black) are used to estimate the transition forces $F_{{\rm tr}}^I$$F_{{\rm tr}}^I$, $F_{{\rm tr}}$$F_{{\rm tr}}$ and $F_{{\rm tr}}^{II}$$F_{{\rm tr}}^{II}$. (**C**) Force versus trap position trajectory during a pull–relax cycle on an ATGC molecule in 5 mM NaCl. During pulling (blue), ATGC exhibits four transitions (a.–d.) The first three transitions display bistability (a. and a′. in inset *ii*, b. in inset *i*) while the fourth transition is irreversible (c. in inset *i*). During relaxation (red), the molecule rehybridizes at about 20 pN (d.). (**D**) Pull trajectories of an ATGC molecule in the presence of 0.5 M glyoxal (1 M NaCl, pH 7.9) before (*t*_0_; blue) and after glyoxal reaction for 30 s (*t*_30s_; green) and 55 s (*t*_55s_; red) while kept in the fully extended state at 74 pN (gray dotted line). The first transition (a.) at about 65 pN (see also a. in panel (A)) occurs at lower forces after glyoxal exposure, at 58 pN after 30 s and at 47 pN after 55 s, respectively.

Figure 4A shows a representative profile of force versus trap position for the ATGC construct in 1 M NaCl. During pulling (blue) the molecule undergoes two distinct transitions (a.) and (b.) at which the construct becomes stepwise longer (decrease in force), and both processes are seen to be fully reversible without any detectable hysteresis during the relaxation (red). The fact that the whole transition is reversible shows that ATGC does not undergo full melting in the investigated force range; a disruption of all base pairs would have resulted in a hysteretic relaxation curve similar to that of single-clicked 5′AT (Figure 2A). During both transitions the ATGC construct is seen to jump between two states of distinct lengths (inset *i.*) indicating that the molecule exhibits a conformational bistability during both steps.

Since the two transitions are almost fully separated (inset *i.*) the total force-induced process in Figure 4A was modeled using three states, adding an intermediate between the B-form at low force and the final extended state at high force (see Supplementary information). The force versus position data for each pull and relax trajectory was assigned to the three states based on the proximity of each data point to linear fits in the transition region (Figure 4A, inset *ii.*). The probability of populating each state was then plotted as a function of force and fitted to the three-state model (see Figure 4B), using the pooled data from all trajectories for the molecule in Figure 4A. The transition force *F*_tr_ for the overall process is defined as the maximum in the fitted population of the intermediate state, resulting in the value 64.3 ± 0.8 pN (*n* = 8). The average total extension of the molecule at *F*_tr_ was measured to be 24.7 ± 1.0 nm (0.202 ± 0.008 nm/bp).

### The extension of ATGC involves both melting and S-DNA formation

The observed extension at *F*_tr_ can be used to exclude the possibilities that the overall transition simply reflects the whole ATGC undergoing a full B-to-S conversion or a full melting, internally or by peeling. Thus, for the ATGC molecule the measured degree of extension at *F*_tr_ implies that it undergoes a combination of melting and B-to-S conversion. In order to investigate how melting is involved in the force-induced transitions in ATGC, we performed stretching experiments with glyoxal added to the buffer, as used to examine 3′5′AT (Figure 3D). Figure 4D shows the results of one such experiment, with pull trajectories before (blue) and after (green, red) a given ATGC molecule in the fully extended state was exposed to glyoxal for different durations (30 s and 55 s). Before being exposed to glyoxal (blue), the molecule undergoes the two transitions seen in Figure 4A. The gradual disappearance of the first transition (a.) with increasing exposure time to glyoxal and a simultaneous decrease in melting force clearly indicate that the first transition involves melting. By contrast, the second transition (b.) is virtually unaffected by the glyoxal, indicating that this transition is mainly a B-to-S conversion as concluded from the same type of glyoxal experiment on the GC-part by itself (23).

Close inspection of the second transition (inset *i.* of Figure 4D) shows that the intermediate state is partly affected by glyoxal, as detected by a slightly shorter transition after exposure (green, red) than before (blue). This observation suggests that the intermediate itself consists of two states with a transition between them. Another indication of a split intermediate state is seen in inset *ii.* of Figure 4A that shows that the linear fit to the intermediate state (green line) has a smaller gradient than the fits to both the initial and final states, whereas a single intermediate state is expected to have a slope that is a linear combination of the two bracketing states. The degree of extension of the intermediate was estimated by measuring the extensions to and from the intermediate state at the transition forces $F_{{\rm tr}}^{\rm I}$ and $F_{{\rm tr}}^{{\rm II}}$ obtained from the fitted three-state model (see Figure 4B) as the crossing points between the population of the intermediate state and those of the B-form and fully stretched state, respectively. The analysis shows that during the first transition the molecule is on average extended (contracted during relaxation) by 8.0 ± 2.1 nm, and during the second transition it is extended by an additional 11.9 ± 1.9 nm. This implies an extension of the intermediate of about 4.8 ± 1.61 nm between $F_{{\rm tr}}^{\rm I}$ and $F_{{\rm tr}}^{{\rm II}}$.

### The effect of ionic strength supports that ATGC first melts then forms S-DNA

Figure 4C shows a representative pull–relax cycle when the ATGC construct is stretched in 5 mM NaCl. In contrast to the two transitions at high salt (Figure 4A) pulling at low salt (blue) results in four transitions, three reversible transitions at about 45, 49 and 59 pN (a., a′. and b.) followed by a fourth irreversible transition (c.). Transition (c.) shows that the construct becomes completely melted at high enough force, as indicated by the ensuing hysteresis during the relaxation curve (red) where the ATGC duplex is not reformed until the force is reduced to about 20 pN as is typical for a fully melted single-clicked construct (Figure 2A and B). Transition (b.) occurs at essentially the same force as transition (b.) in Figure 4A, indicating that it is due to a B-to-S transition in the GC-part (23). By contrast, the transition (a.) is strongly affected by the lower ionic strength, and splits into two transitions (a.) and (a′.) both at lower forces than transition (a.) at high salt. This sensitivity to ionic strength supports that transition (a.) indeed involves melting, in view of the similar effect of salt on the 5′AT construct (Figure 2). Also, the split into two melting transitions is in agreement with the hypothesis that the intermediate state observed at high salt (Figure 4A) is a composite of two states where the duplex is further melted/partly rehybridized between them (transition a’. in Figure 4C).

## DISCUSSION

The results on the 5′AT construct show that the force-induced melting of a designed AT-rich sequence can be investigated systematically in a short DNA duplex by including a covalent end-link between the strands in the synthesis. The force response of the same base sequence in the two constructs 3′5′AT and ATGC will be discussed by comparing to the 5′AT molecule.

### 5′AT

The results of Figure 2 indicate that the AT sequence studied here melts under tension, as supported by previous results when its high-force state was probed by glyoxal (23). The hysteresis during the rehybridization indicates that the melted state is peeled where one strand is completely released from the duplex so that the applied force is carried by the second strand itself. As a result, the opposing bases will come out of register and can only pair up when the applied force has been reduced substantially, to about 20 pN, so that the extension of the force-carrying strand becomes similar to the opposite, relaxed strand. The force distributions of the melting and rehybridization reactions are wide (Figure 2D), but the results still clearly show that both forces decrease when the ionic strength is decreased as expected from stronger electrostatic strand–strand repulsion. Secondly, the melting of the present AT-rich sequence involves an intermediate state at both high and low ionic strength (c. in Figure 2A and B), probably a partly peeled molten helix, since the intermediate returns to the B-form (the force-distance seen branch at low force) after each jump, at least at the current 1 kHz measuring frequency.

### 3′5′AT

That the 3′5′AT construct undergoes a continuous transition when the force is increased (Figure 3) while 5′AT melts irreversibly (Figure 2) is most likely explained by the presence of a second linker in 3′5′AT preventing the duplex from melting by one strand peeling off from the other, so that the melting instead occurs through a process where a bubble of non-paired bases is nucleated internally in the duplex (7). The constructs 3′5′AT and 5′AT thus provide two molecules with the same base sequence where these two principal melting mechanisms can be studied. The gradual melting of 3′5′AT indicates a non-cooperative transition, and the good fit in Figure 3B indicates that internal melting can be described as a continuous transition between two states, B-form and a fully melted 3′5′AT molecule. The abrupt melting of the B-form 5′AT could not be described by such a model. The melting-force distribution for 3′5′AT (Figure 2D *iii.*) is as wide as for 5′AT, but at a given ionic strength (5 mM NaCl) 3′5′AT melts at a significantly higher force than for 5′AT, in agreement with that its melting temperature also at zero force is higher (23).

A second difference between the two modes of force-induced melting concerns how the applied tension is distributed between the two strands. In the case of peeling, only one strand carries the tension (the other strand is relaxed), whereas in the case of internal melting the tension is shared between both strands (Figure 1A). This difference implies that the transition length going from the duplex to either of the two molten states should be different in 5′AT and 3′5′AT, with internal melting resulting in a shorter transition.

Thirdly, the 3′5′AT and 5′AT constructs allow us to compare how peeled and internally molten states undergo rehybridization. Although both constructs are completely molten at high force, 5′AT exhibits hysteresis at both low and high ionic strength (Figure 2A and B), while 3′5′AT tends to rehybridize reversibly, especially at high salt (Figure 3A). That both strands are under tension may explain why 3′5′AT tends to rehybridize reversibly because the linkers keep the two strands in close proximity while in 5′AT they are not necessarily aligned perfectly base to base since one of the strands is relaxed. More experiments are needed to explain why an internally molten molecule (3′5′AT) may rehybridize by both mechanisms, reversibly (Figure 3A) or with hysteresis (Figure 3C).

### ATGC

The equilibrium force ($F_{{\rm tr}}^{\rm I}$, *P* = 0.5) of the first transition in the ATGC construct (62.1 ± 1.06 pN) (Figure 4B) agrees with the melting force measured for the single-clicked 5′AT construct in 1 M NaCl (61.5 ± 2.62 pN). When the salt concentration is lowered, the melting force for the 5′AT is reduced by ∼18 pN (Figure 2C), a behavior that is shared by the first transition in the ATGC-dimer (Figure 4C). These results show that the low-force transition in the ATGC construct is due to the AT-rich region melting via strand peeling. The second transition in ATGC has a much weaker salt dependence, which supports that it corresponds to a B-to-S conversion because our previous studies of the GC-rich monomer by itself shows that the conversion force to S-DNA is much less sensitive to ionic strength (23). Taken together, the observations at high and low salt (Figure 4A and C) show that the ATGC construct first melts reversibly via peeling of the AT-rich part in two steps (a. and a’.), followed by a B-to-S transition (b.) in the GC-rich part when the force is further increased. This interpretation is supported by the glyoxal experiments on the ATGC sequence (Figure 4D), which show that only the first transition is markedly affected by glyoxal and thus involves melting.

The difference between the observed two melting processes in the 5′AT and the ATGC construct, where the first one is predominantly irreversible and the second one completely reversible, is most likely explained by differences in the molten states. In the 5′AT sequence, the two strands are kept in proximity by the covalent linker when the region is fully melted. However, although the strands are held close to each other, the bases are not likely to be aligned. By contrast, in the case of the ATGC construct the two strands are held together by the still base-paired flanking GC region that not only keeps the two separated strands in close proximity to each other but also keeps at least the closest bases in register. This arrangement allows the two molten strands to re-zip much easier and will thus increase the rehybridization rate. We also note that formation of any stable secondary structures within the peeled strand is likely to cause significant hysteresis during relaxation. In anticipation of this potential problem, one of the design criteria for the AT-core sequence used here was that it should not form any stable hairpins under the experimental conditions.

## CONCLUSIONS

That the ATGC construct first undergoes melting in the AT-part as the force is increased and then a B-to-S transition in the GC-part indicates that the two types of force-induced processes that we observe in the separate AT- and GC-parts are additive. This observation suggests that the force-behavior of long DNA molecules may be understood by studying short designed duplexes that exhibit the force-induced processes in a pure form, and then use them as building blocks to form longer, more complex DNA molecules. In such a strategy, an important question is whether the results obtained with click-linked synthetic oligonucleotides are representative of native DNA. Our previous results show that the linkers do not affect the B-to-S transition as the clicked GC sequence overstretches in the same manner as the native GC molecule does (23). In the case of melting, comparison with a linker-free reference sequence is inconclusive because the irreversible loss of the tether between the two beads caused by melting may also reflect a broken bead attachment. However, in the ATGC construct the melted AT sequence is prevented from falling apart because of the stabilizing flanking GC-part that forms S-DNA under tension instead of melting (23).

The construct 3′5′AT shows how important the base composition is for determining whether a certain DNA molecule will form S-DNA or melt under tension. Even though the thermal melting of the AT-rich sequence is significantly inhibited by the two inter-strand linkers in 3′5′AT (23), under tension this construct prefers a molten conformation instead of the base-paired S-form. The lack of cooperativity in the melting (Figure 3A) suggests that the initial melting bubbles are only a few bases in size but also that the sequence melts without passing through the S-form. The melting of the AT-part in ATGC shows that a neighboring sequence prone to form S-DNA has little inhibiting effect on melting.

The lack of hysteresis when the AT sequence is covalently sealed at both ends (Figure 3A) and the reversible peeling observed in the ATGC construct (Figure 4A) both suggest that the largest barrier to overcome during rehybridization is the formation of the first base pairs. Our findings agree well with the recently published results of King *et al.* and Zhang *et al.* and may shed some light upon the noted differences regarding internal melting in the two studies (14,18). Zhang *et al.* report that internal melting is a hysteretic transition that was not observed by King *et al.* Our results show that at least for the short molecules studied here, both internal melting as well as peeling can be non-hysteretic. The two studies also showed differences regarding which transition type occurred at given, identical salt concentrations. The authors suggested that the observed differences could be due to local heating by the optical traps in the King *et al.* study, shifting the equilibrium toward melting. However, we observe melting of the AT sequence at even higher salt concentrations and furthermore estimated the heating effect in our setup (see Supplementary information) to have negligible effect on our measurements. The authors also suggested that an alternative explanation for their observed differences could be sensitivity to variation of DNA sequence, which against this background appears more plausible. An important conclusion of this study is that while hysteresis is a sign of melting, a lack of hysteresis does not necessarily mean that the molecule does not melt. Indeed, segments of DNA exhibiting fast non-hysteretic melting, mixed with segments undergoing B-to-S transition, could also explain some variations between Zhang *et al.* and recent results from the Lombardi group (38).

## SUPPLEMENTARY DATA

Supplementary Data are available at NAR Online.

## FUNDING

European Research Council; King Abdullah University of Science and Technology; Chalmers Library; Biotechnology and Biological Sciences Research Council [BB/J001694/1 sLoLa to T.B. and A.H.E-S.].

*Conflict of interest statement*. None declared.

## Supplementary Material

SUPPLEMENTARY DATA
