# Supplementary material for: Force-induced melting of DNA—evidence for peeling and internal melting from force spectra on short synthetic duplex sequences
Source: Nucleic Acids Res. 2014 May 16;42(12):8083–91. doi: 10.1093/nar/gku441 (PMC4081069; doi:10.1093/nar/gku441)
Supplement: SUPPLEMENTARY DATA [file supp_42_12_8083__index.html]

Force-induced melting of DNA—evidence for peeling and internal melting from force spectra on short synthetic duplex sequences — Force-induced melting of DNA—evidence for peeling and internal melting from force spectra on short synthetic duplex sequences — SUPPLEMENTARY DATA 

# Force-induced melting of DNA—evidence for peeling and internal melting from force spectra on short synthetic duplex sequences

## SUPPLEMENTARY DATA

**Files in this Data Supplement:**

- SUPPLEMENTARY DATA
