# Supplementary material for: Force-induced melting of DNA—evidence for peeling and internal melting from force spectra on short synthetic duplex sequences
Source: Nucleic Acids Res. 2014 May 16;42(12):8083–91. doi: 10.1093/nar/gku441 (PMC4081069; doi:10.1093/nar/gku441)
Supplement: SUPPLEMENTARY DATA [file supp_gku441_nar-00042-f-2014-File006.pdf]

Supplementary Information

Analyzing the reversible transitions of ATGC using a three state model. Using the following procedure the force vs. trap position data of Figure 4A are assigned to three different states. The initial low-force “B” state and the high-force fully stretched state “S” are first assigned by the proximity of the data points to lines fitted below and above the transition, respectively. The line describing the intermediate state “I” is then seeded as the average of the low- and high-force lines. Finally all three lines are refitted to include the assigned data points.

The two transitions in ATGC are then described using a non-degenerate three state model. The probabilities of finding the molecule in any of the three states are assumed to follow the Boltzmann distribution (assuming pseudo-equilibrium during pulling and relaxation) so that the population probabilities of the three states (P_B, P_I, and P_S) are given by

$$P_B = \frac{1}{q} \tag{S1}$$
$$P_I = \frac{e^{-\Delta E_{B\to I}/kT}}{q} = \frac{e^{-(\Delta G_{B\to I} - F\Delta x_{B\to I})/kT}}{q}$$
$$P_S = \frac{e^{-\Delta E_{B\to S}/kT}}{q} = \frac{e^{-(\Delta G_{B\to S} - F\Delta x_{B\to S})/kT}}{q}$$

where $\Delta G_{B\to I}$ and $\Delta G_{B\to S}$ are the differences in free energy at zero force between the B-form and the intermediate I and the S-state, respectively, $\Delta x_{B\to I}$ and $\Delta x_{B\to S}$ the corresponding cooperative lengths during the transitions, and F the applied force. The sum over states q is given by

$$q = 1 + e^{-(\Delta G_{B\to I} - F\Delta x_{B\to I})/kT} + e^{-(\Delta G_{B\to S} - F\Delta x_{B\to S})/kT} \tag{S2}$$

with the degeneracy of all three states assumed to be unity.

Force versus position measurements. The stretching and relaxation of the molecules are performed by changing the position of the trap at a constant rate of 50 nm/s on the average, achieved by moving the trap in steps of 1 nm approximately every 20 ms. Since the time it takes for a typical molecule to convert from one state to another is a millisecond or faster, the trap can be treated as stationary during the transition. An effect of the protocol used here is that transitions between states of different lengths are observed as a change in force. Extension of the molecule is observed as a drop in force as the bead relaxes towards the center of the trap, and a contraction as an increase in force since the bead is pulled further away from the trap center. For a given position interval of the trap within the transition region, the probability of being in a state can thus be measured as the relative time spent in that state. However, while the trap position is a useful measure of the extent of the transition in a molecule it is not directly comparable between molecules. To that end we map a certain trap position to a force (binned at 0.5 pN) which then can be compared between different molecules. The conversion between trap position and force is made using the average of the lines fitted to the “B” and “S” states (see Fig. 4A inset ii. blue and red lines, respectively) for the first pull cycle of each molecule. The binned data are pooled for all pull and relax cycles of a molecule and the probabilities are then calculated as the relative time spent in each state at a given force interval. The model has four parameters, $\Delta G_{B\to I}, \Delta G_{B\to S}, \Delta x_{B\to I}, x_{B\to S}$ in equation (S1). In order to increase the robustness of the overall fit the probabilities of the “B” and “S” states were first fitted separately using a two state model. The parameter estimates from those fits were then used as starting points when fitting the full three state model. The transition force F_{tr} is defined as the maximum value of P_I, and is given by

$$\frac{\partial P_I}{\partial F} = 0 \to F_{tr} = \frac{\ln\left(\frac{\Delta x_{B\to I}}{\Delta x_{B\to S} - \Delta x_{B\to I}}\right) + \Delta G_{B\to S}}{\Delta x_{B\to S}}$$

Analyzing the reversible transitions of 3'5'AT using a two state model. A similar type of analysis using two states is performed to describe the transition observed for the double-clicked AT-rich construct at 1 M NaCl. However, as we are unable to discern discrete steps within the transition a different measure of the extent of the transition must be used. Here, this is provided by the relative distance of a data point from the initial low force state. Lines are fitted to the Force vs. Trap position data at forces above and below the transition to define a "B" and "S"-state, respectively. As before, due to the relatively slow stepwise movement the trap can be considered to be stationary during the transition so that fluctuations in the length of the molecule are observed as changes in force. Binning of the data is therefore performed as described above, and the relative distance is calculated for each interval as the normalized mean difference in force of the data relative to the "B"-state. The relative distance is then plotted as a function of force (mapped using the average of the "B"- and "S" fits) and fitted to a two state model. The transition force F_{tr}, at which the transition length is measured, is taken as the force where the relative distance is 0.5.

Instrument setup. Figure S3 shows a schematic outline of the optical tweezers instrument. The output from two counter-propagating 150 mW, 845-nm diode lasers are used to form a single trap within a fluidics chamber mounted on a motorized stage. The fluidics chamber is divided into three channels; two of the channels are used to dispense the two types of coated polystyrene beads into the third main channel where the experiments are performed. Integrated into the main channel is a glass micropipette used to immobilize one of the beads by sucking it onto the tip. The light from the lasers is guided through single-mode optical fibers to so-called "fiber wigglers", that control the position of the formed trap in the plane (x, y) perpendicular to the optical axis (z). In the wigglers, the optical fiber is passed through a set of brass tubes and comes out in the other end through a grid which acts as a pivoting point for the protruding fiber. Piezoelectric actuators are used push on and bend the optical fiber to reposition the trap to the desired location. The cone of light that emanates from the tip of the fiber is passed through a pellicle beam splitter that redirects about 5% of the intensity onto a position-sensitive detector (PSD) giving a current that is proportional to the x-y position of the trap.

The remaining light is focused through a water-immersion objective (60x, NA 1.20) to form the trap in the fluidic chamber. The light exiting the trap is collected by an identical objective lens and redirected to both a PSD and a photodiode (PD) for force measurement in the x-y plane and along the z-axis, respectively. Redirection of the light exiting the condensing objective lens to the force detectors is achieved using polarizing beam-splitters and two quarter-wave plates to turn the polarization of the light 90° relative to the light entering focusing objective lens. When a force is exerted on a bead in the optical trap, the distribution of the refracted light hitting the PSDs changes and gives to a change in the signal current that proportional to the force. The photodiodes measuring the force along the z-axis are made sensitive to the distribution of the light exiting the trap by a filter with a circular transmission profile which attenuates the light based on the size of the beam. The force measurement approach relies on the conservation of light momentum, which means that the calibration is independent of factors such as size and shape of the trapped bead, buffer viscosity, or laser power.

The analog current signals from the detectors are digitized into 16-bit signals and processed by PIC microcontrollers in the electronics of the instrument before the data is sent to a computer where it is converted into positions and forces. The microcontrollers also handle time critical tasks of the instrument control, such as force and position feedback. Further information about the instrument setup can found in refs. (35, 36, 38) and at http://tweezerslab.unipr.it/.

Calibration. The experimental setup measures force by detecting the change in the light momentum entering and exiting the optical trap (36,38). The force acting on the trapped bead is proportional to the measured detector signal by a factor M as

$$F = M * \Delta\text{PSD}$$

where ΔPSD is the detector signal relative to a zero-force reference. For the experimental setup used here, it can be shown that the calibration factor M solely depends on the physical characteristics of the instrument (36,38).

$$M = \frac{R_d}{c\Psi R_l}$$

Here, R_d is the half-width of the position sensitive detector (PSD), c the speed of light, Ψ the power sensitivity of the detector and R_l the focal length of the objective lens. The effective dimensions (R_d) and response linearity of the PSDs were measured by scanning the detectors using a test laser (Melles Griot, 830 nm, 8 mW) mounted on the motorized xyz-stage of the instrument. The sensitivity, Ψ (ADC-signal/watts), was measured using an optical power meter (Thorlabs, PM30).

The determined calibration factor was then tested using Stokes' law ($F=3\pi\eta dv$) by moving the fluidics chamber attached to the motorized stage at different velocities around a trapped polystyrene bead. The test was performed in distilled water at room temperature (22.3 °C, viscosity taken as η=9.48*10^{-4} Pa s) for 50 beads with a by the manufacturer reported mean diameter of 2.1±0.051 µm (Spherotech, Inc; IL USA). A linear regression of the measured force vs velocity data resulted in an estimated mean diameter of the sampled beads of 2.08±0.10 µm, suggesting that the force calibration is accurate to within 1 %. The measured difference between the two methods is similar to what has previously been reported for this type of instrument setup (39).

Since the force is proportional to a change in the detector signal (ΔPSD) relative to set point corresponding to zero-force, an error in the set point will result in an offset in force and increase the variance in a series of measurements. The detector values corresponding to zero force is set for each molecule prior to formation of the tether between the two beads when the bead is free to relax within the trap. Determination is performed by averaging the force over 32 ms with all flows in the fluidics chamber turned off. The accuracy of the determined zero force value is coupled to factors that affect the thermal motion of the trapped bead and thus varies with temperature, bead size and trap stiffness. For the reported data set the standard deviation of the zero-force set point was 0.10 pN, evaluated by measuring the force variance over several seconds after the tether between the two beads was broken.

The influence of drift in the force measurements was evaluated using the change of the zero-force set point in a series of consecutive measurements and showed an average drift 0.003 pN/min. This type of analysis gives an estimate of the drift on a time scale ranging from minutes to hours, but agrees well with dedicated measurements performed on the same and shorter time scales. With a mean total duration of an experiment on an individual molecule of 2 min 35 s, the estimated drift suggest an error due to drift in force on the order of 10^{-2} pN.

The wiggler and the trap position PSDs are calibrated against the shaft encoders of the actuator motors (Thorlabs Z-606) driving the motorized stage. The calibration procedure is performed by following a bead immobilized on the pipette with the optical trap using a constant force feedback protocol. The stage and the micropipette are then moved using the actuator motors, and the wigglers follow by repositioning the trap in order to keep the force constant. The calibration factors for the position PSDs are then determined by relating the recorded detector signal to the movement of the motors. The position drift during the reported experiments was up to a few nm/min, but the effect of this is significantly reduced due to a relatively fast pulling rate and that each pull- and relax cycle is analyzed individually.

Estimation of local heating due to trap light absorption. Absorption of the light forming the optical trap can lead to a local increase in temperature at the focus. The described experimental setup measures the temperature outside of the fluidics chamber and therefor records a temperature that, due to this effect, is destined to be lower than that at the trap focus. To evaluate the potential influence that local heating may have on the measurements we here estimate the increase in temperature at the focus. In a study by Mao et al. (41), the local heating induced by a focused 975 nm laser beam was measured and modeled in an instrument setup very similar to ours (including fluidics chamber). Their results showed that the heating increased the temperature at the focus by 5.6°C/100 mW which agreed with the results from their analytical model based on the Lambert-Beer law.

$$\Delta T(r, r') = \frac{P_{in}\alpha_W}{2\pi K} ln\frac{r'}{r}$$

The expression shows that the difference in temperature ΔT decays logarithmically with increasing distance from the focus, r, as it approaches a cut-off distance r'. It also shows that increase in temperature is proportional to the power of the light, P_{in}, and the absorption of the medium, α_W. The absorbed heat is balanced by the thermal conductivity, K. In our setup the power used to form the trap is about 100 mW, and has a wavelength of 845 nm at which the absorption of water is ~13 times lower than at 975 nm (42). Performing the calculation for our setup yields an increase in temperature at the focus of the trap by ~0.5°C. The estimate suggests that absorption of the trapping light is only likely to have a limited effect on the experimental results in this study.

References

36. Bustamante, C. and Smith, S.B. (2007) United States patent no. US 7,274,451 B2.
37. Smith, S.B., Cui, Y. and Bustamante, C. (2003) Optical-trap force transducer that operates by direct measurement of light momentum. *Methods Enzymol.*, **361**, 134-160.
39. Bustamante, C. and Smith, S.B. (2006) United States patent no. US 7,133,132 B2.
40. Huguet, J.M., Bizarro, C.V., Forns, N., Smith, S.B., Bustamante, C. and Ritort, F. (2010) Single-molecule derivation of salt dependent base-pair free energies in DNA. *Proc. Natl. Acad. Sci. U. S. A.*, **107**, 15431-15436.
41. Mao, H., Ricardo Arias-Gonzalez, J., Smith, S.B., Tinoco, I., Jr. and Bustamante, C. (2005) Temperature Control Methods in a Laser Tweezers System. *Biophys. J.*, **89**, 1308-1316.
42. Palmer, K.F. and Williams, D. (1974) Optical properties of water in the near infrared. *J. Opt. Soc. Am.*, **64**, 1107-1110.

5'AT & 3'5'AT

```
5'-XACCTAGTTTCATTAAGAGATGTCGTAAAATTCTTATTACAATAGTTTAGCCTGACAGAAATXA   OligoAT

5'-YAATTTCTGTCAGGCTAAACTATTGTAATAAGAATTTTACGACATCTCTTAATGAAACTAGGYA   ATComplement


5'-XACCTAGTTTCATTAAGAGATGTCGTAAAATTCTTATTACAATAGTTTAGCCTGACAGAAATXA
   ||||||||||||||||||||||||||||||||||||||||||||||||||||||||||||||
3'-AYGGATCAAAGTAATTCTCTACAGCATTTTAAGAATAATGTTATCAAATCGGACTGTCTTTAAY

OligoAT: #A 23, #G 9, #C 9, #T 21, #X 2, 64-mer (GC: 28.1%)
ATComplement: #A 23, #G 9, #C 9, #T 21, #Y 2

3'5'AT: X = OCTADINYL dU, Y = AZIDOHEXANOYLAMINO C6 dT.
5'AT: 3'-X of OligoAT and 5'-Y of ATComplement are replaced by T.
```

ATGC

```
5'-CCTAGTTTCATTAAGAGATGTCGTAAAATTCTTATTACAATAGTTTAGCCTGACAGAAATATCA
ACAGAGCCACTTTGGCCCGCTGGTCGCCGCTATCGACAGGCTCAATGCTGGAGGGTXA          OligoATGC

5'-YAACCCTCCAGCATTGAGCCTGTCGATAGCGGCGACCAGCGGGCCAAAGTGGCTCTGTTGATAT
TTCTGTCAGGCTAAACTATTGTAATAAGAATTTTACGACATCTCTTAATGAAACTAGG          ATGCComplement


5'-CCTAGTTTCATTAAGAGATGTCGTAAAATTCTTATTACAATAGTTTAGCCTGACAGAAATATCA ...
   ||||||||||||||||||||||||||||||||||||||||||||||||||||||||||||||||
3'-GGATCAAAGTAATTCTCTACAGCATTTTAAGAATAATGTTATCAAATCGGACTGTCTTTATAGT ...

...ACAGAGCCACTTTGGCCCGCTGGTCGCCGCTATCGACAGGCTCAATGCTGGAGGGTXA
   ||||||||||||||||||||||||||||||||||||||||||||||||||||||||||
...TGTCTCGGTGAAACCGGGCGACCAGCGGCGATAGCTGTCCGAGTTACGACCTCCCAAY

OligoATGC: #A 34, #G 27, #C 27, #T 33, #X 1, 122-mer (GC: 44.3%)
ATGCComplement: #A 34, #G 27, #C 27, #T 33, #Y 1

X = OCTADINYL dU, Y = AZIDOHEXANOYLAMINO C6 dT.
```

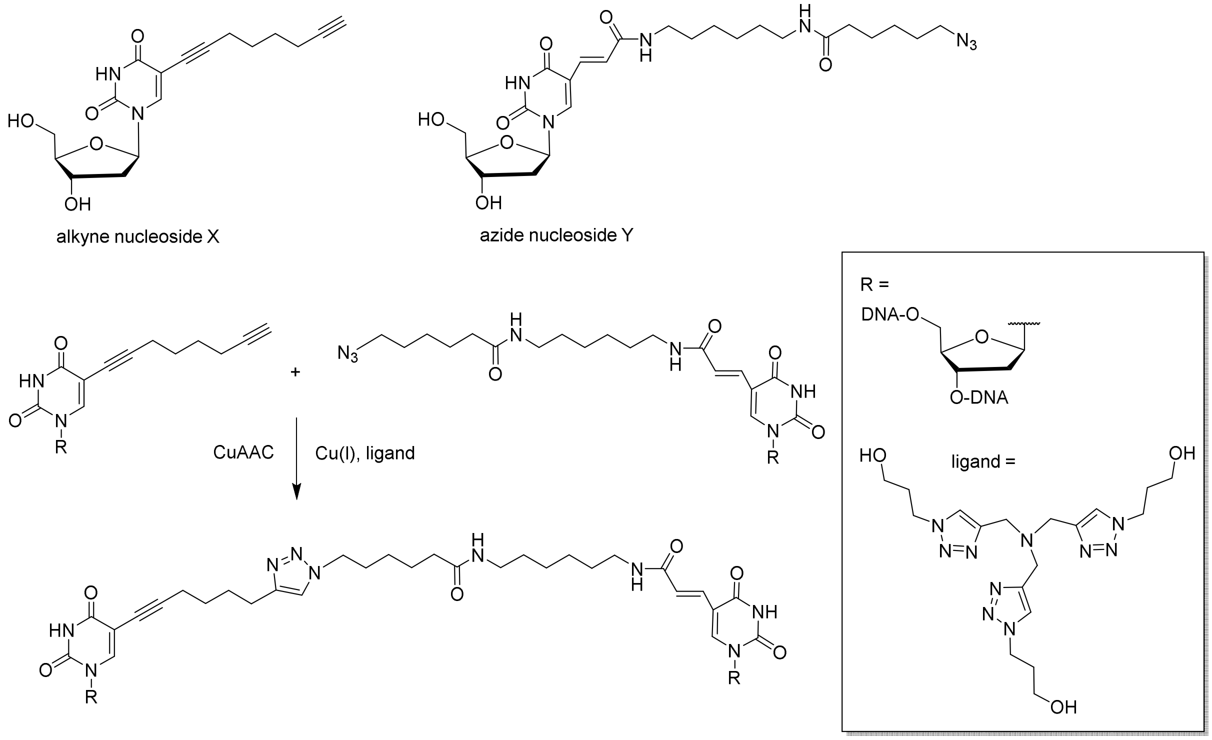

Figure S1. Base sequence of the oligonucleotide duplex regions, modified nucleosides, and scheme of the CuAAC click reaction.

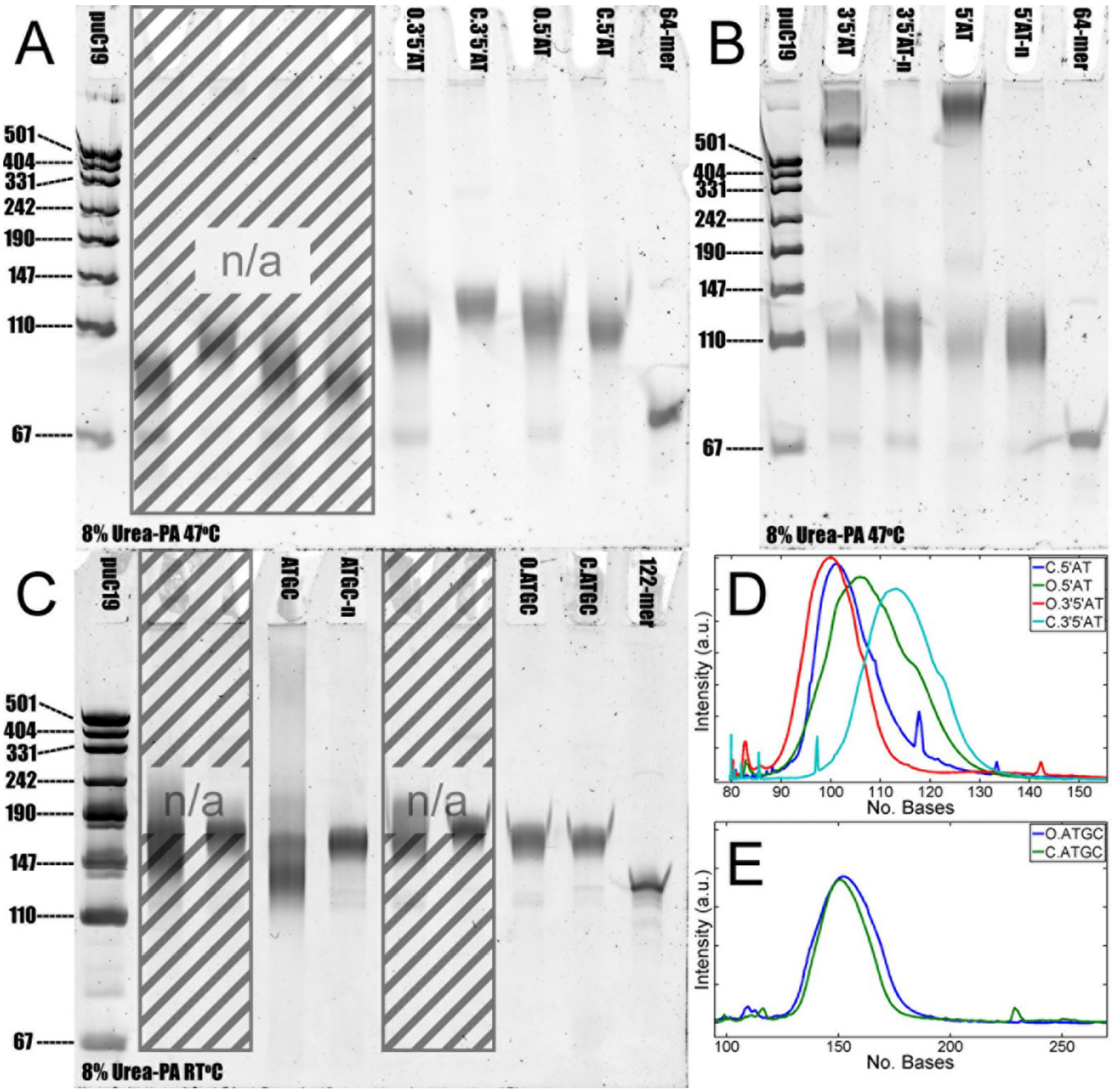

Figure S2. Evaluation of terminal transferase extension and CuAAC click reaction using denaturing polyacrylamide gel electrophoresis. Sample compositions are given in Table S1. (A) 8% denaturing (7 M urea) polyacrylamide gel run at 47 °C showing the results of the terminal transferase extension reaction of the single-stranded oligonucleotides and their complements for the two AT constructs. (B) 8% denaturing (7 M urea) polyacrylamide gel showing the results of the CuAAC click reaction for the single- and double-clicked AT constructs. Under these conditions the double-clicked 3’5’AT construct has a melting temperature between 34 – 41 °C and the single-clicked 5’AT a melting temperature of about 30 °C. The gel is run at 47 °C and shows how the melted clicked constructs (3’5’AT & 5’AT) are retarded compared to the non-clicked (3’5’AT-n & 5’AT-n) constructs which are fully dissociated into their single stranded components. The double-clicked 3’5’AT sample exhibits two distinct bands corresponding to the desired product and products where only one of the two inter-strand links are formed and thus had to be purified. (C) 8% denaturing (7 M urea) polyacrylamide gel run at room temperature (22 °C) showing the results of the terminal transferase extension and CuAAC click reaction for the ATGC construct. (D) Lane profiles of the terminal transferase extended ss-oligonucleotides in (A). The results show an extension of about 40 - 50

nucleotides based on the puc19 size reference. (E) Lane profiles of the terminal transferase extended ss-oligonucleotides in (C). The analysis shows that the formed handles are each about 30 nucleotides in length.

Table 1. Sample descriptions for polyacrylamide gels in Figure S2.

Lane Label	Description
puC19	Size marker puC19/MspI (HpaII) (Fermentas SM0221)
O.3’5’AT	Terminal transferase extended OligoAT (see Fig. S1) with two click sites
C.3’5’AT	Terminal transferase extended ATComplement (see Fig. S1) with two click sites
O.5’AT	Terminal transferase extended OligoAT (see Fig. S1) with one click site
C.5’AT	Terminal transferase extended ATComplement (see Fig. S1) with one click site
64-mer	OligoAT, 64 b (see Fig. S1)
3’5’AT	Clicked 3’5’AT construct before purification
3’5’AT-n	Nonclicked 3’5’AT construct
5’AT	Clicked 5’AT construct
5’AT-n	Nonclicked 5’AT construct
ATGC	Clicked ATGC construct
ATGC-n	Nonclicked ATGC construct
O.ATGC	Terminal transferase extended OligoATGC (see Fig. S1)
C.ATGC	Terminal transferase extended ATGCComplement (see Fig. S1)
122-mer	OligoATGC, 122 b (see Fig. S1)

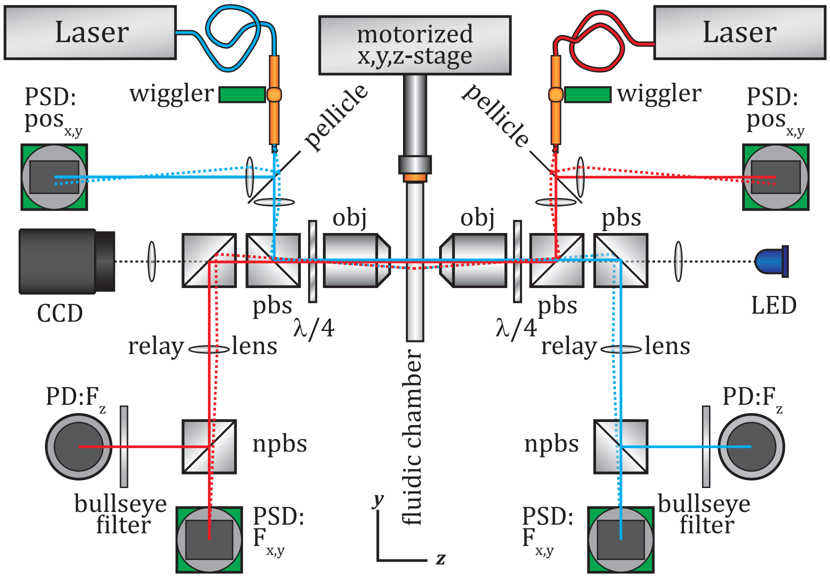

Figure S3. Schematic representation of the experimental setup. Two 845 nm 150 mW fiber coupled diode lasers (Lumics GmbH) are used to form a single trap in a fluidic chamber mounted on a motorized (Thorlabs, Z-606) stage (Newport Corp., 561D-XYZ). The lasers are connected to wigglers, which are devices that control the position of the trap within the fluidic chamber. The output from the wigglers is passed through a pellicle beam-splitter which redirects about 5% of the light to a PSD (OSI Optoelectronics, DL-4) where the position of the trap is recorded. The remaining light is redirected using a polarizing beam-splitter (pbs), and passed through a quarter-wave plate (λ/4) before being focused by a water-immersion objective lens (Olympus, 60x, NA 1.2) to form the trap. The light exiting the trap is collected by an identical objective lens and passed through a second quarter-wave plate. The light exiting the second plate has a polarization that is perpendicular relative to its original linear polarization which allows the optical paths of the two lasers to be separated and the exiting light to be redirected for detection. A relay lens is used to reimage the back-focal-plane of the objective lens onto both a PSD (OSI Optoelectronics, DL-10) and a photodiode (PD, OSI Optoelectronics, PIN-10DI) for force measurement in the x-y plane and along the z-axis, respectively. The light is divided between the force detectors using a non-polarizing beam-splitter (npbs) and before the light hits the photodiode it is passed through a graduated “bullseye” filter to radially attenuate the beam so that the force signal along the z-axis can be scored properly. One of the objective lenses is mounted on a linear stage which allows positioning of it along the z-axis to focus the two beams in the same plane.
